# A framework for seroepidemiologic investigations in future pandemics: insights from an evaluation of WHO’s Unity Studies initiative

**DOI:** 10.1186/s12961-023-00973-z

**Published:** 2023-05-16

**Authors:** Karen Hennessey, Lorenzo Pezzoli, Carsten Mantel

**Affiliations:** MMGH Consulting, Zurich, Switzerland

**Keywords:** COVID-19, SARS-CoV-2, Seroepidemiologic investigations, Pandemic preparedness

## Abstract

**Background:**

The WHO Unity Studies initiative supports countries, especially low- and middle-income countries (LMICs), in conducting seroepidemiologic studies for rapidly informing responses to the COVID-19 pandemic. Ten generic study protocols were developed which standardized epidemiologic and laboratory methods. WHO provided technical support, serological assays and funding for study implementation. An external evaluation was conducted to assess (1) the usefulness of study findings in guiding response strategies, (2) management and support to conduct studies and (3) capacity built from engagement with the initiative.

**Methods:**

The evaluation focused on the three most frequently used protocols, namely first few cases, household transmission and population-based serosurvey, 66% of 339 studies tracked by WHO. All 158 principal investigators (PIs) with contact information were invited to complete an online survey. A total of 19 PIs (randomly selected within WHO regions), 14 WHO Unity focal points at the country, regional and global levels, 12 WHO global-level stakeholders and eight external partners were invited to be interviewed. Interviews were coded in MAXQDA™, synthesized into findings and cross-verified by a second reviewer.

**Results:**

Among 69 (44%) survey respondents, 61 (88%) were from LMICs. Ninety-five percent gave positive feedback on technical support, 87% reported that findings contributed to COVID-19 understanding, 65% to guiding public health and social measures, and 58% to guiding vaccination policy. Survey and interview group responses showed that the main technical barriers to using study findings were study quality, variations in study methods (challenge for meta-analysis), completeness of reporting study details and clarity of communicating findings. Untimely study findings were another barrier, caused by delays in ethical clearance, receipt of serological assays and approval to share findings. There was strong agreement that the initiative created equitable research opportunities, connected expertise and facilitated study implementation. Around 90% of respondents agreed the initiative should continue in the future.

**Conclusions:**

The Unity Studies initiative created a highly valued community of practice, contributed to study implementation and research equity, and serves as a valuable framework for future pandemics. To strengthen this platform, WHO should establish emergency-mode procedures to facilitate timeliness and continue to build capacity to rapidly conduct high-quality studies and communicate findings in a format friendly to decision-makers.

## Background

On 30 January 2020, the novel coronavirus, severe acute respiratory syndrome coronavirus 2 (SARS-CoV-2), responsible for coronavirus disease 2019 (COVID-19) was declared a public health emergency of international concern by WHO. As is the case with any epidemic of a novel pathogen, decision-makers were challenged to determine the appropriate public health and social measures (PHSM) with limited information on the virus transmission characteristics, extent of infection, pathogenesis and scale of the threat.

Responding to this knowledge gap and building on lessons and existing study protocols from influenza, Middle East respiratory syndrome-related coronavirus and Zika virus epidemics, WHO launched the Unity Studies initiative. The aim of the initiative is to support countries, especially low- and middle-income countries (LMICs), to conduct SARS-CoV-2 seroepidemiologic investigations designed to rapidly inform national and global public health responses to COVID-19 [1].

The cornerstone of the initiative was to have easily accessible generic protocols providing standardized epidemiologic and laboratory methods for conducting SARS-CoV-2 seroepidemiologic investigations. This facilitated comparability across studies, allowing them to be combined for informing regional and global pandemic response strategies. A total of 10 generic protocols were developed, including (1) the first few cases and their contacts (FFX), (2) household transmission (HHT), (3) population seroprevalence (SEROPREV), (4) pregnancy outcomes and transmission, (5) school transmission, (6) surface contamination, (7) health facility transmission (two protocols) and (8) vaccine effectiveness (VE) (two protocols). The protocols were translated into five languages (Arabic, Chinese, French, Russian and Spanish).

Along with establishing study protocols, the Unity Studies initiative (1) facilitated or provided technical assistance on protocol adaptation, study implementation, data analysis and reporting, (2) fully or partially funded studies in LMICs, (3) led data dissemination efforts to make study findings publicly available and (4) organized learning opportunities and knowledge exchange through scientific webinars and regional seminars. Technical support was provided for ethical clearance of protocols, especially for WHO-supported Unity Studies, which required WHO Research Ethics Review Committee (ERC) approval in addition to locally acquired ERC approval.

Countries adopting Unity Studies protocols have access to laboratory support including (5) advice on selecting serologic test kits, (6) provision of serologic test kits free of charge to LMICs and (7) serology panels for validating test kits. After evaluating available immunologic diagnostic tests, guidance was issued for WHO Unity Studies to use the Wantai total antibody enzyme-linked immunosorbent assay (ELISA) as the preferred test kit.

WHO used its three organizational levels by assigning Unity Studies focal points at the headquarters (HQ), regional offices (RO) and country offices (CO) to manage, create awareness and support study implementation. Investigators from LMICs could seek Unity Studies support by having their study protocol reviewed, and if designated as Unity-aligned (based on methodological alignment, local ethical approval and agreement to share findings), they became eligible to receive funds and laboratory support.

The initiative established technical partnerships with Epiconcept, SeroTracker and the University of Melbourne, and operational partnerships with the Africa Centres for Disease Control and Prevention, the European Centre for Disease Prevention and Control (ECDC), the Global Outbreak Alert and Response Network (GOARN), Pasteur Institute and United States Centers for Disease Control. In addition, fundraising efforts yielded major financial resources, including the COVID-19 Solidarity Response Fund (approximately US$ 5 million) and the German Federal Ministry of Health COVID-19 Research and Development Funds (approximately US$ 9 million).

As of 22 September 2021, WHO had information on 339 Unity-aligned studies (WHO-supported and non-WHO-supported) that were planned, ongoing or completed, in 114 Member States in all six WHO regions. The most frequently adopted of the 10 protocols were the SEROPREV, with 149 studies (44% of all studies); the FFX and HHT studies with 76 studies (22% of all studies); and health worker cohort and case–control studies with 60 studies (18% of all studies).

This report describes findings from a WHO HQ-commissioned external evaluation of the Unity Studies initiative conducted by MMGH Consulting (MMGH). The evaluation was to gather insights from Unity Studies principal investigators (PIs) and stakeholders and assess what worked well and what could be optimized in terms of preparedness and capacity to conduct or facilitate timely seroepidemiologic investigations in future large-scale outbreaks. The specific objectives were to assess (1) the usefulness of study findings in informing national, regional and global COVID-19 response strategies, (2) management of the initiative and the provision of support to Unity Studies and (3) research capacity built from engagement with the initiative (Table 1).

**Table 1:** Overview of the evaluation’s analytic variables and information management

Content areas and variables	Purpose	No. of areas or variables	Description of areas or variables (categories)
Evaluation objectives	To provide an overall framework for the evaluation	3	A. Usefulness of findings for informing COVID-19 policies and communications B. WHO management, technical support areas C. Capacity built from being part of the initiative
Survey and interview groups	To seek a variety of insights using different methods	4	A. Online survey—PIs B. Interview group—PIs C. Interview group—WHO staff D. Interview group—external partners
A priori thematic areas	To use to code and manage interview data	12	See Fig. 1
Stratification variables	To use for analysis of survey data and PI interviews	4	A. WHO region (six WHO ROs^a^) B. Income level (LMIC or HIC^b^) C. Study type (SEROPREV, FFX/HHT or multiple^c^) D. Receipt of WHO support (yes/no)
Categories of usefulness of study findings^d^	To quantify insights on usefulness provided from the survey and interviews	3	A. Informing PHSM policies B. Informing vaccination policies C. Knowledge generation and communication
Categories of support areas and management^d^	To describe and quantify insights on support areas	4	A. Technical assistance B. Laboratory support, serologic assays C. Funding D. Management (coordination, human resources, administrative processes, data-sharing)

^a^RO for Africa (AFRO); RO for the Americas (AMRO); RO for the Eastern Mediterranean (EMRO); RO for Europe (EURO); RO for South-East Asia (SEARO); RO for the Western Pacific (WPRO)

^b^HIC, high-income country

^c^Multiple represents when a PI has led a combination of studies including either FFX or SEROPREV or both

^d^The survey variables used a 5-point Likert scale; interview responses were coded as “positive”, “mixed” or “negative”

**Fig. 1:**
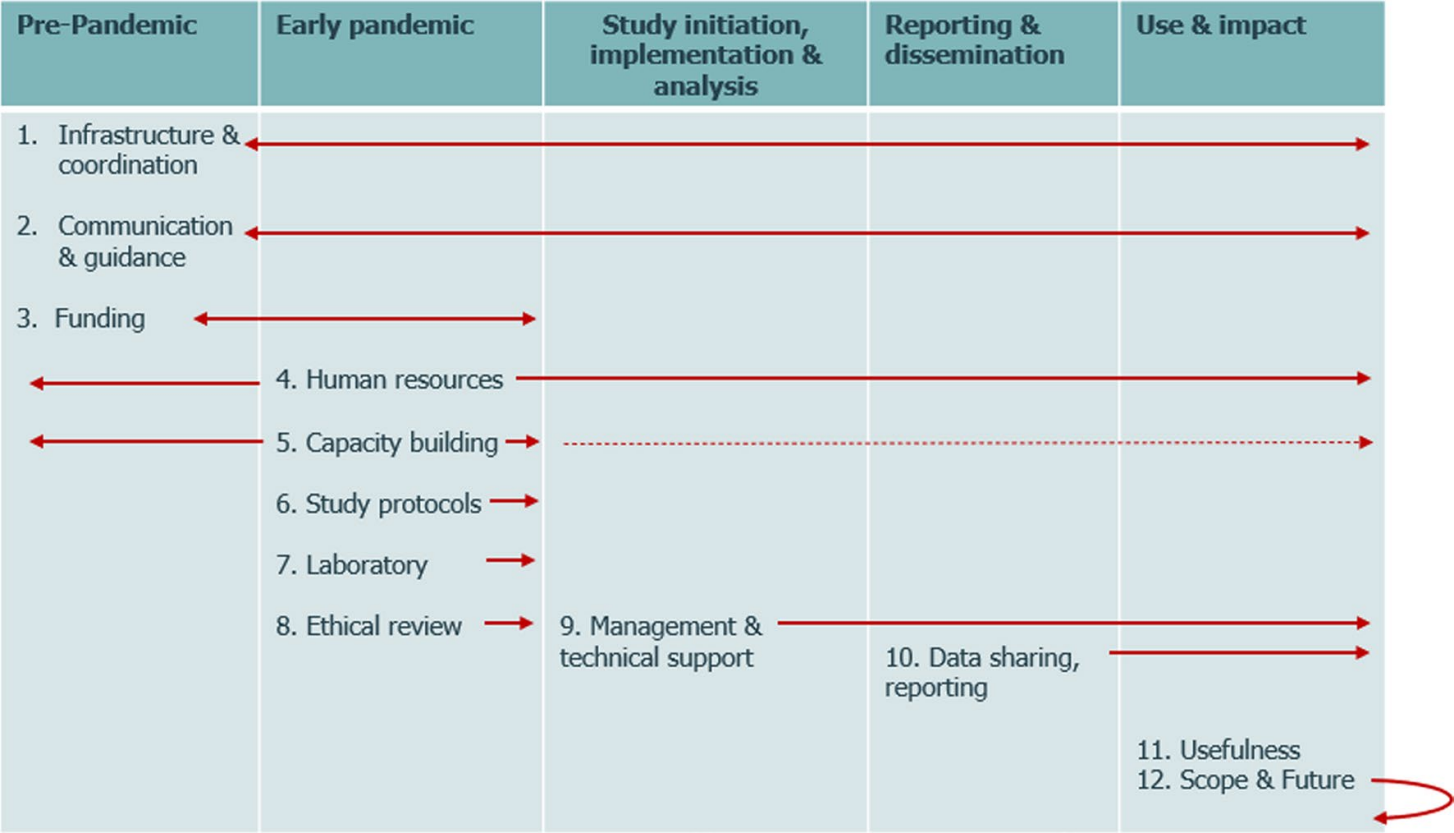
Twelve thematic evaluation areas used for coding interview data, shown in the context of pandemic and Unity Studies stages

## Methods

A guiding principle of the evaluation was to implement an approach that would lead to timely, practical and actionable feedback. To facilitate this, the evaluation focused on the three most commonly adopted protocols: FFX, HHT and SEROPREV. Due to the similarity of the FFX and HHT protocols (both are designed to investigate all identified close contacts of laboratory-confirmed COVID-19 cases to characterize the transmission dynamics and clinical spectrum of SARS-CoV-2 infection), these were combined in the analysis and reporting of findings. This evaluation included (1) an online survey of Unity Studies PIs and (2) interviews with several groups including study PIs, Unity Studies focal points and stakeholders at all levels of WHO, and external partners (Table 1).

### Survey of Unity Studies PIs

Of the 225 FFX, HHT and SEROPREV studies tracked as of 22 September 2021, 193 had unique PIs, and among these, 156 studies (81%) had PI contact information (e.g. some studies supported by partner agencies provided study details but lacked PI contact information) and were invited to participate in the survey. The survey covered the evaluation’s three specific objective areas and was formulated to rank performance in each area. It was designed to be completed within 10 minutes and was tested by the project team. The final survey had 20 categorical and open-ended questions and was implemented using the Qualtrics™ survey platform (www.qualtrics.com).

Starting in October 2021, PIs were informed of the survey by WHO and were subsequently sent a personalized online link to complete the survey by MMGH. Nonresponders were sent two automatic reminders and one personal reminder from the evaluation team. The survey closed at the end of December 2021.

### Interview groups

#### Unity Studies PIs

Nineteen (12%) studies were included for PI interviews. Ten of them were selected in proportion to the number of studies per WHO region, and nine were purposefully selected from each region, based on feedback from RO Unity focal points on studies that could yield valuable lessons, both positive and negative.

#### WHO staff

All four WHO HQ Unity Studies focal points, all six RO focal points and six WHO CO focal points from the 19 countries with PI interviews (one selected from each region) were identified for interviews. Twelve key WHO HQ COVID-19 stakeholders were identified for interviews. These included senior emergency programme management and COVID-19 Incident Management Support Team (IMST) staff, laboratory and data platform focal points, and managers of COVID-19-related WHO programmes including Solidarity II (a global collaboration led by WHO promoting a broader scope of SARS-CoV-2 serological surveys).

#### Unity Studies external partners

Persons from all eight of the initiative’s technical and operational partners were identified to be interviewed.

### Conduct of interviews

A checklist, composed of 14 questions related to the evaluation’s specific objectives and aligned with the survey tool, was used to guide 30-minute interviews. The interviews took place using the Microsoft Teams online platform. Interviews were conducted by senior MMGH staff. Verbatim notes were taken during the interviews. Immediately following the interviews, notes were cleaned and organized to facilitate qualitative analysis and reporting.

### Analysis

#### Quantitative analysis

Survey respondents and nonrespondents were compared for differences in the distribution of the four stratification variables shown in Table 1. Means for scaled questions were calculated by assigning five points to the most positive response and one point for the most negative response. Chi-square tests and odds ratios were calculated to measure differences in categorical data. Positive feedback from scaled questions was defined as “extremely useful” and “very useful” for usefulness questions and as “excellent” and “good” for support-related questions. Interview segments were directly coded as positive, negative or mixed. Analysis of responses related to usefulness excluded studies where PIs reported that usefulness was not known or not known yet.

#### Qualitative analysis

Segments of interview notes were coded to manage content and conduct qualitative analyses using MAXQDA™. A priori thematic areas were established to help organize and code interview notes (Fig. 1, Table 1). Other codes were added as common themes emerged. For each evaluation category, a three-order translation table was generated using interview segments as the first order, synthesis into findings as the second order and conclusions as the third order [2, 3]. The translation tables were cross-validated by two of the evaluation team members. To preserve confidentiality, presentation of exemplary interview quotes does not distinguish HQ Unity Studies focal points and HQ stakeholders.

## Findings

### Description of participants

#### PI survey respondents

A total of 69 PIs (44%) completed the survey. Survey respondents and nonrespondents (*n* = 87) were similar with regard to stratification variables (Table 1), except PIs were more likely to respond if they had WHO support and were from an LMIC and less likely to respond if from the WHO European Region (Table 2).

**Table 2:** Comparison of survey respondents and nonrespondents

Stratification variable	Strata	No. (%)		Odds ratio (95% CI)
		Survey respondents (*n* = 69)	Survey nonrespondents (*n* = 87)	
WHO RO	AFRO	28 (41%)	25 (29%)	Reference
	AMRO	8 (12%)	6 (7%)	1.19 (0.36–3.91)
	EMRO	6 (9%)	10 (11%)	0.54 (0.17–1.69)
	EURO	9 (13%)	23 (26%)	0.35 (0.14–0.89)
	SEARO	8 (12%)	7 (8%)	1.02 (0.32–3.22)
	WPRO	10 (14%)	16 (18%)	0.56 (0.21–1.45)
Income level	HIC	10 (14%)	27 (31%)	Reference
	LMIC	59 (68%)	60 (69%)	2.66 (1.18–5.67)
Study type	SEROPREV	40 (58%)	61 (70%)	Reference
	FFX/HHT	20 (29%)	17 (20%)	1.76 (0.83–3.77)
	Mix	9 (13%)	9 (10%)	1.5 (0.55–4.11)
WHO support	No	27 (39%)	59 (68%)	Reference
	Yes	42 (61%)	28 (32%)	3.38 (1.69–6.34)

AFRO, RO for Africa; AMRO, RO for the Americas; EMRO, RO for the Eastern Mediterranean; EURO, RO for Europe; SEARO, RO for South-East Asia; WPRO, RO for the Western Pacific

The 69 respondents represent studies conducted in 52 countries; 61 (88%) were conducted in LMICs. Seventy percent of respondents reported having fully completed the study. There was no difference in study completion rates between regions. Reasons provided for not completing studies (*n* = 21) included delays in local ethics or government approvals, limited human resource capacity, delays in obtaining laboratory kits, late or insufficient funding or that the study had only recently started.

#### Interviewees

Interviews were completed for 15 (83%) of the 19 studies selected for PI interviews, four of six WHO CO Unity Studies focal points, all six of the RO focal points, all four of the WHO HQ focal points and all 12 of the WHO identified stakeholders. Among the 15 PIs interviewed, 11 had led SEROPREV studies, 13 had conducted their studies in LMICs, at least one was from each of WHO’s six regions and nine had completed their studies at the time of interview.

### Using study findings for guiding policies and communication

#### Informing PHSM polices and strategies

Among PI survey respondents, 65% reported that findings were useful for informing or reinforcing PHSM polices and strategies, with a mean score of 3.8 (Table 3). When comparing the responses by income level, LMIC PIs ranked usefulness higher than high-income country (HIC) PIs (mean scores of 3.9 and 3.1, respectively; distribution in scaled responses, *X*^2^ = 11.9, *p* < 0.02). There were no other differences when comparing usefulness scores within the stratification variables.

**Table 3:** PIs’ feedback on Unity Studies evaluation areas

Evaluation area	Topic	On-line survey (*n* = 69)			Interviews (*n* = 15)	
		No.^a^	Mean score	% Positive feedback	No.^a^	% Positive feedback
Usefulness^b^	Informing PHSM policies	55	3.8	65	12	69
	Informing vaccination policies	52	3.7	58	6	50
	Knowledge generation and communications	61	4.3	87	12	75
Support areas^c^	Technical	43	4.4	95	12	92
	Financial	39	3.9	59	11	55
	Laboratory	43	4.0	56	10	30

^a^Studies were excluded if a PI reported that usefulness was not known or not known yet

^b^When narrowing to only SEROPREV studies (*n*=36), % positive feedback increases to 64%, mean usefulness 3.9; among PI interviews, all six responses were from SEROPREV studies

^c^Mean scores average the combination of timeliness and adequacy

Among PI interviewees, 69% reported that study findings were useful for informing or reinforcing PHSM (Table 3).

At least one interviewee from each of the interview groups (Table 1) provided positive insights on the usefulness of study findings for informing or reinforcing policies and strategies. Exemplary quotes are shown below.

*The combination of results of the three studies gave a good idea of prevalence of infection, which was useful in some ways for planning. Study results were immediately shared with the Emergency Coordination Team and with the Ministry decision-makers*. (PI FFX/HHT)

*The MoH [Ministry of Health] was able to use findings to guide policy decisions, for example to adapt testing policies so they focus on specific subpopulations.* (PI SEROPREV)

*Studies provided a substantial contribution—especially seroprevalence studies. Preliminary results were presented to government officials to use for decision-making—on how the government will calibrate PHSM.* (WHO CO)

*Unity Studies have played an important role in informing decision-making. The information provided by Unity Studies has fed into understanding who are the priority groups for interventions. This includes vaccination polices and PHSM (e.g. school policies).* (WHO RO)

*SEROPREV data can help adjust vaccination strategies to country settings, especially in LMICs*. (WHO HQ)

*FFX impacted decisions in country X and country Y, driving policies about quarantine, testing strategies, etc. They were early in the pandemic and needed information*. (Partner)

At the same time, having delayed results was the most frequently reported barrier to using data for guiding polices. Exemplary quotes are shown below.

*It was unfortunate that the results came late. We could have done better to inform decision-making on time. It would have been beneficial for the response*. (PI SEROPREV)

*Delay and subsequent usefulness was a concern for all of us. The value of the study would have been bigger at the beginning*. (PI SEROPREV)

*Data took an entire year to collect. Today the data we have on the original variant may no longer be relevant*. (PI FFX/HHT)

*The challenge with conducting seroprevalence studies is that by the time the data is out there, it is outdated and not so useful as it could be in real time.* (WHO RO)

*Results of seroprevalence studies are mostly too late for being able to influence policy—timeliness is a major issue of limiting use of data*. (WHO HQ)

*They [FFX] did not produce findings quick enough and the situation evolved quickly, suddenly there were so many cases.* (Partner)

Concerns with study quality (methods, implementation and interpretation) may have limited confidence in study findings and been a barrier to using them. Exemplary quotes are shown below.

Concerns with methods and implementation:

*Especially at the beginning, not all protocols were implemented “carefully”*. (WHO RO)

*They were taking too many shortcuts with the FFX/HHT protocols…* (WHO HQ)

Challenges and limitations with interpreting data:

…*information produced from seroprevalence studies was very “noisy” and prone to misuse/misinterpretation*. (WHO RO)

*Interpretation of the data could have been better*. (WHO HQ)

In terms of using study findings at the regional and global levels, feedback was mixed, but focused predominantly on challenges with combining studies for meta-analyses and limited data triangulation and discussion on the implication of study findings on regional and global COVID-19 policies. Exemplary quotes are shown below.

Positive feedback on usefulness of guiding regional and global policies:

*Unity Studies is a clear confirmation that virus was travelling everywhere. This knowledge was particularly important to our overall strategies*. (WHO HQ)

*The data has been helpful. We see high seroprevalence in Africa and it is good to have this information*. (WHO HQ)

Challenges with comparing and combining studies for meta-analysis:

*The biggest problem is statistical representation in seroprevalence studies. Which areas are selected, which populations? It makes the data very heterogeneous*. (WHO HQ)

*At the beginning it was brilliant that Unity was deploying the same test kit at the start—it provided comparability. This is not the case anymore. Assays are different and they measure different things, answer different questions*. (WHO HQ)

*Despite the Unity approach, there are still many countries that have different approaches (using different kits, age groups) making global decision-making a challenge*. (WHO RO)

*The main challenge is that there is a huge amount of variation in study methodology and implementation. The Unity Studies protocol helps in this sense, at the same time contextual data must be taken into consideration*. (Partner)

*Through the review for meta-analysis, we found a lot of differences in how protocol aspects were interpreted. For example, different studies (cultures) have different definitions of a household member. The protocol needs to be generic, but lack of clear definitions led to variations*. (Partner)

*The biggest bottleneck relates to inconsistent quality control indicators. There is an unevenness in the way these studies are implemented. For example, in country X, they used different lab cutoffs and did not provide reasons for doing so*. (Partner)

Limited data triangulation and discussion on the implications of findings:

*There are no mechanisms for discussing findings for decision-making and policy-setting at the regional level*. (WHO RO)

*There is limited discussion about the implications of the findings for the region or globally*. (WHO RO)

*More extensive and comprehensive elaboration of results at the higher levels is needed*. (WHO HQ)

*Triangulation with surveillance data missing*. (WHO HQ)

*We need an analysis of Unity Studies data together with other data complementing this information. …to give value to the data even if scarce, and to put these in a broader context*. (WHO HQ)

#### Informing vaccination polices and strategies

Among PI survey respondents, 58% reported that findings were useful for informing or reinforcing vaccination polices and strategies, with a mean score of 3.7 (Table 3). There were no differences when comparing usefulness scores within the stratification variables. Similarly, among PI interviewees, 50% said that study findings were useful for informing vaccination policies (Table 3) and specified that they were used to guide planning, identify hotspots and prioritize populations for vaccination.

Among interviews with RO focal points, one region reported that SEROPREV data were presented and used for decision-making among national immunization technical advisory groups (NITAGs); there were no other reports of using findings to guide vaccination policies at the regional level. At the global level, several interviewees reported that using Unity Studies findings to guide vaccination policies was limited because other data (e.g. from COVID-19 surveillance and modelling) were used for target-setting. A few interviewees remarked that vaccination experts were not integrated early enough into the WHO HQ IMST structure and that the WHO Strategic Advisory Group of Experts on Immunization (SAGE) was only recently engaged with the UNITY initiative and discussing its findings.

#### Knowledge generation and communications

Among PI survey respondents, 87% reported that findings were useful for knowledge generation and communications, with a mean score of 4.3 (Table 3). LMIC PIs ranked such usefulness higher than HIC PIs (mean scores of 4.4 and 3.8, respectively; distribution in scaled responses, *X*^2^ = 10.0, *p* < 0.05). There were no other differences when comparing usefulness scores within the stratification variables.

Among PI interviewees, 75% reported that study findings were useful for informing COVID-19 communications and contributing to knowledge generation (Table 3). At the country level, there was generally good dissemination of findings to government officials. Multiple interviewees reported that study findings were communicated via websites, national television and social media. One PI reported that findings were useful for combating rumours and misinformation. Interestingly, there were a few countries that withheld findings of higher-than-expected seroprevalence from the public, for fear of derailing PHSM or vaccination drives.

At the RO level, there were mixed experiences, with some ROs leading highly valued and regular seminars to share data and experiences, while others lacked the capacity to do so. At the HQ level, several partnerships were formed, such as with GOARN (a WHO network that supports the prevention and control of infectious disease outbreaks and public health emergencies) to help compile updated lists of Unity Studies for regular dissemination, with SeroTracker to update, grade risk of bias of individual studies and visualize Unity Studies findings on a web platform [4] and with Zenodo to share prepublished findings. Despite these efforts, multiple interviewees felt that another layer of analysis was needed to position findings in a broader context to increase the understanding and use of study findings. Exemplary quotes regarding feedback on knowledge generation and communications are shown below.

Positive insights:

*Then MoH provided general information every day to the public, Dr X was periodically directly giving updates on national TV. The public wants to listen to scientists directly, and she has thus become a famous person in country X*. (PI SEROPREV)

*We are using data in official communications—speaking a lot about it. More should be using these seroprevalence data in Executive Board or World Health Assembly meetings*. (WHO HQ)

*It [SEROPREV data] is a proxy for capturing the number of cases—since we cannot always have case data. We are using data in official communications—speaking a lot about it.* (WHO HQ)

*[For example] Unity data show us that we are nowhere near having herd immunity. We still have a lot of people at risk. From a global advocacy perspective, Unity data is very powerful*. (WHO HQ)

*From a global perspective the biggest value is that it has been giving us a better view of cases reported and real incidence. We can get a sense of the under-ascertainment*. (WHO HQ)

…*Unity data are shared with the modellers too. …they are shared for communication between countries. Countries are very interested in knowing how they are doing compared to their neighbours*. (WHO RO)

*Early on, SEROPREV data were published in newspapers before being on preprint. FFX and HHT results were made available early on, to back up PHSM*. (Partner)

*Another example of the global use is comparing LMIC and HIC, or situations where we have both anti-spike and natural infections. These are very useful data to understand COVID-19 epidemiology*. (Partner)

*Seroprevalence data is crucial to understand the burden of past infection and how this impacts the epidemiology going forward (e.g. herd immunity).* (Partner)

Negative insights:

*We are tracking thousands of studies, reviewing risks of bias. But more can be done so they can be used more. We need better articulation, not necessarily more, but better*. (WHO HQ)

*What was missed in terms of using findings is the translation aspect in communications*. (WHO HQ)

*I have not seen a lot of public communications coming out from Unity, so I cannot comment much on this. Internally, in WHO, there has been a lot of visibility*. (WHO HQ)

*More could be done to work with WHO COs to support use of results. Some of the results were never released.* (WHO HQ)

*There is not a systematic communication strategy to disseminate findings*. (WHO RO)

### Unity Studies support areas and management

#### Technical support

Technical support provided by the Unity Studies initiative was very highly appreciated, with 95% of the PI survey respondents and 92% of interviewees providing positive feedback (Table 3). Technical support was provided systematically as part of protocol review, laboratory considerations and ethical clearance, followed by more ad hoc support on study implementation, analysis and reporting, provided by the Unity Studies team and technical partners via a help desk. Exemplary quotes are provided below.

*Great support from the Unity Secretariat with adapting the protocols in countries and with technical review*. (WHO RO)

*Technical assistance is really high-level. Governments do not have strong epidemiologists. The lab technical support was also appreciated*. (WHO RO)

*We got a lot of advice on sample size and on the adjusting for the clustered design in the analysis. It was timely and adequate.* (PI SEROPREV)

*Technical support was provided very quickly from the Unity Secretariat Team. Each time the study team had questions the secretariat organized a meeting very quickly. They provided a lot of support for the protocol writing*. (PI SEROPREV)

The availability of study protocols was highly valued across all interview groups, with specific feedback provided on how they saved time by having a solid starting point and facilitated international comparability. There was mixed feedback on flexibility of protocols, with some interviewees appreciating this while others stated that such flexibility delayed implementation. One RO focal point noted that it could take a few months of back and forth to finalize the protocols. There were requests for core variables to be established as part of the protocol, for more detail on sampling strategies and for updating protocols over time as pandemic characteristics evolved.

Ethical review processes and experiences varied across countries and regions, and the global level. Many countries did not have issues with local clearance because of existing mechanisms for expedited reviews, and their ERCs were experienced in reviewing operational research protocols. If ROs had an ERC in place, WHO ethical review took place at the regional level; otherwise, it was at the HQ level. Feedback indicates that the process was much smoother at ROs than at the HQ. The global-level ethical clearance process came with substantial challenges, including that WHO HQ ERC engagement changed over time, feedback was often delayed due to infrequent convening, and assessments were made more along the lines of clinical trials than through the lens of operational research. Several interviewees questioned the added value of WHO HQ ethical clearance on top of local ERC clearance. One area that was reported to be helpful for facilitating local ERC approval was the WHO HQ preapproval of the generic FFX protocol.

Regarding data management support, some interviewees complained that the data collection and analysis software that was provided, Go.Data [5], required too much time to learn how to use it and was not similar to what countries and investigators were using or had used in the past. Some PIs wished there was more support for data analysis and were interested in having more engagement with other colleagues on analytic approaches. Exemplary quotes are shown below.

*It is important to help colleagues with the validation of the data, which is probably needed, given the capacity of their teams is limited in terms of number of staff*. (Partner)

*One issue was the people engaged. Sometimes they did not have the technical expertise in that domain (or enough background on mathematics and statistics to handle this type of research).* (Partner)

*Go data was made available, but nobody wanted to use it. It is not a familiar tool and could not be adopted in time. Timing is important, and the middle of pandemic is not the time for adopting something new*. (Partner)

*It would be important to have processes for the systematic validation of study findings to ensure that the protocol is followed correctly and to support countries with the analysis and interpretation of findings*. (WHO RO)

#### Financial support

Among PI survey respondents who received financial support, a little more than half (59%) were pleased with the support received (Table 3). Similarly, among PI interviewees, six of the 11 gave positive feedback on funding received through WHO. Some reported that WHO funds had made the difference in being able to conduct their studies. Some negative feedback was related to adequacy, timeliness and short expiry periods of funds. Partial funding sometimes served as a catalyst, but searching for funds to fill gaps delayed implementation. WHO HQ feedback concurred by indicating that SEROPREV studies were expensive, and it was difficult to cover the entire costs for the larger ones. More financial support would have allowed for more representative samples in some studies. There were some complaints about complex WHO procedures, timelines, awarding criteria, and the administrative paperwork.

#### Laboratory support

There were many positive reports on the value of WHO HQ in providing guidance on serologic test kits and facilitating test kit validation. However, among the initiative’s support areas, laboratory support scored the lowest. Slightly more than half (56%) of the PI survey respondents were happy with laboratory support, compared to only three of the 10 PIs interviewed. This discrepancy might have been due to PIs being more comfortable or spontaneous in speaking about pitfalls during the interview sessions (Table 3).

Low satisfaction was mainly due to the delayed receipt of test kits. Even though only one serologic kit was found to perform well enough to be considered a preferred test kit for Unity Studies, the delays were reported to be associated with procurement and distribution, and not a supply issue on the manufacturing side. The process of evaluating and identifying preferred test kits involved independent validations which were conducted rapidly but showed inconsistencies due to the use of different evaluation methodologies. Sorting out these differences increased the length of time until the preferred test kits could be made available.

Several interviewees would have liked more in-country support to ensure procedures were well implemented and more engagement and discussion on validating serologic kits and laboratory methods. ROs and COs stated that their laboratory capacity was limited, while at the same time some ROs would have wanted to be more involved in making decisions about test kit recommendations (e.g. preferred quantitative vs qualitative assays, less time-consuming automated assays), even though choices are limited at the beginning of an outbreak of a novel pathogen.

Exemplary quotes are shown below.

Positive insights:

*The lab experts were on the ground but needed some guidance. Unity filled that need*. (WHO RO)

*The support in general for lab aspects was very much appreciated*. (WHO RO)

*We had several discussions on laboratory technical issues regarding what we plan to do. They were very timely and adequate*. (PI SEROPREV)

*From the lab side, the study team appreciated the support and capacity built to implement ELISA tests*. (PI SEROPREV)

*They received training on laboratory aspects also in terms of equipment. It was helpful to process a vast number of samples*. (PI SEROPREV)

Negative insights, suggestions:

*It would have been very interesting to not just send a matrix for test kit validation (serology panel), but to have teams to check whether we were implementing it well. The ongoing technical validation process would have been good before each report.* (PI FFX/HHT)

*A frustration was the type of test. We would have preferred a qualitative test to meet the demand to know antibody levels, not just yes or no. We would not have chosen these tests.* (PI SEROPREV)

*For laboratory aspects, HQ organized a few lab expert meetings. However, the impression is that use of diagnostic tests requires more support*. (PI SEROPREV)

*More discussion of laboratory issues would've been welcome. Instead, it appeared that there was an indication about which test to use with no space for discussing. Also, there was not much discussion about quality assurance and validation of these kits.* (WHO RO)

*More resources for standardization and validation of kits at country level is needed*. (WHO RO)

*The process of selecting tests was handled by the HQ team with little involvement of RO experts, including on topics on how to organize the lab work. There was not a lot of space for technical discussion in terms of adapting methods, compared to the epidemiologic aspects of the studies*. (WHO RO)

#### Management

Several interviewees commented on how the initiative was an excellent example of working across the three levels of WHO to support study implementation in countries. While such vertical coordination appeared to have worked well, coordination across programmes at WHO HQ and with some partners was questioned. Exemplary quotes are shown below.

*Other epidemics had been differently managed. Here, no one had the whole picture, as the middle layer of analysis was missing. Data from each of the pillars [groups responsible for different aspects of the pandemic response] went directly up the hierarchy without any cross-fertilization across the pillars*. (WHO HQ)

*The coordination pillar is still lacking to make sure all parties are involved—we need to work as a team. For example, in describing estimates of seroprevalence and how the virus is evolving—we need one strong voice, we need to work collaboratively*. (Partner)

There was almost unanimous agreement by WHO and partners that the initiative was significantly understaffed, and the successes achieved were due to extraordinary efforts of those involved, especially in the WHO HQ Secretariat. Designating focal points at ROs and COs was essential for engaging and supporting studies in-country. Uptake and timeliness of Unity Studies was reported to improve considerably once the RO Unity focal points were identified. In addition to overstretched staff, interviewees reported that WHO staff hiring procedures were slow and not operating in emergency mode, staff turnover was high due to short contracts or assignments, and consultants were not always able to perform tasks at hand. In terms of technical expertise, several ROs and COs reported gaps in laboratory science, data analytics and communications. Exemplary quotes are shown below.

*The secretariat was under-resourced to manage this. It is worth designating more support for it. This would help to streamline anything that has to do with timelines and getting the data out*. (WHO HQ)

*The contracting issues were problematic. On average it took 6 weeks. There were supposed to be emergency standard operating procedures (SOPs) for recruitment, but it never went smoothly*. (WHO HQ)

*Money was there, but staffing was insufficient*. (WHO HQ)

*A main bottleneck was that the Unity initiative was “hard to do” for WHO because of limited human resources*. (Partner)

A common theme that emerged was the arduous and time-consuming WHO publication clearance process that delayed the release of technical updates to regions and countries, as illustrated in the following quote:

*Publication of guidance documents faces the same issues. The pace is horrendously slow. There is a misunderstanding about what is at stake here. This is undermining WHO and the organization’s capacity to convene and coordinate*. (WHO HQ)

Feedback on sharing data or findings with WHO differed between interview groups. PIs largely reported no issues with sharing data and had a good level of trust in WHO. However, data-sharing agreements were not consistently in place, and one PI reported that the lack of a formalized data-sharing agreement added unnecessary workload. A few PIs reported that instructions for sharing data were unclear or not communicated from the beginning. From the WHO HQ perspective, timely data-sharing is a concern, with some countries and some partners being said to keep data until publication. WHO RO respondents commented that not all countries were open to sharing, especially HICs. Exemplary quotes are below.

*Data-sharing—we did not talk about that from the beginning, data needs, format, etc. We did not get instructions on this until the end. Data-sharing should have been put in place and we should have weekly meetings and reports*. (Partner)

*And on the output side—need to support getting data online as quickly as possible and into the hands of government decision-makers. Sharing data even if not published. Academics may feel they need to protect data and keep it until it is published. We need to get in the mindset of doing both at the same time: share data early with decision-makers and publish it for the scientific community*. (WHO HQ)

*It would be better if data- or results-sharing were enforced, like the International Health Regulations*. (WHO HQ)

### Capacity-building

In terms of capacity that was built from being engaged with the initiative, 48 (70%) of the PI survey respondents reported an increase in research capacity, 48 (70%) in collaboration or partnerships, 39 (57%) in enhanced surveillance, 37 (54%) in laboratory-related competency and 32 (46%) in scientific writing. Much of the research capacity built came from the technical support provided by the initiative and was seen as a contribution to research equity. All interview groups had almost exclusively positive feedback on capacity-building, especially in terms of the value of the scientific writing workshops and creating platforms for sharing findings and experiences. Exemplary quotes are provided below.

…*from Unity we had capacity-building on the epidemiologic methods side including sample size adjustment of our analysis. We gained new knowledge. We also learned a lot from the data collection exercise*. (PI SEROPREV)

*This allowed us to use the available indigenous capacity of scientists and researchers in the country. At the local levels, capacity-building of state- and district-level officials became possible*. (PI SEROPREV)

*I appreciate the main things—including scientific writing and peer-to-peer support for many countries—I give them credit. Not only on the methodology side but for building capacity.* (WHO HQ)

*Capacity-building initiatives were well received, especially in scientific writing and analysis*. (WHO RO)

*A critical mass of expertise and knowledge was available in the region and beyond… a good foundation for a future pandemic and for countries for their own surveillance programmes (measles, hepatitis).* (Partner)

*The initiative was good in building research networks and capacity in view of future pandemics. Such competence-building was not done by the usual suspects, there were lots of other countries (LMICS) which were contributing as well*. (Partner)

### Unity Studies’ biggest value

**Fig. 2:**
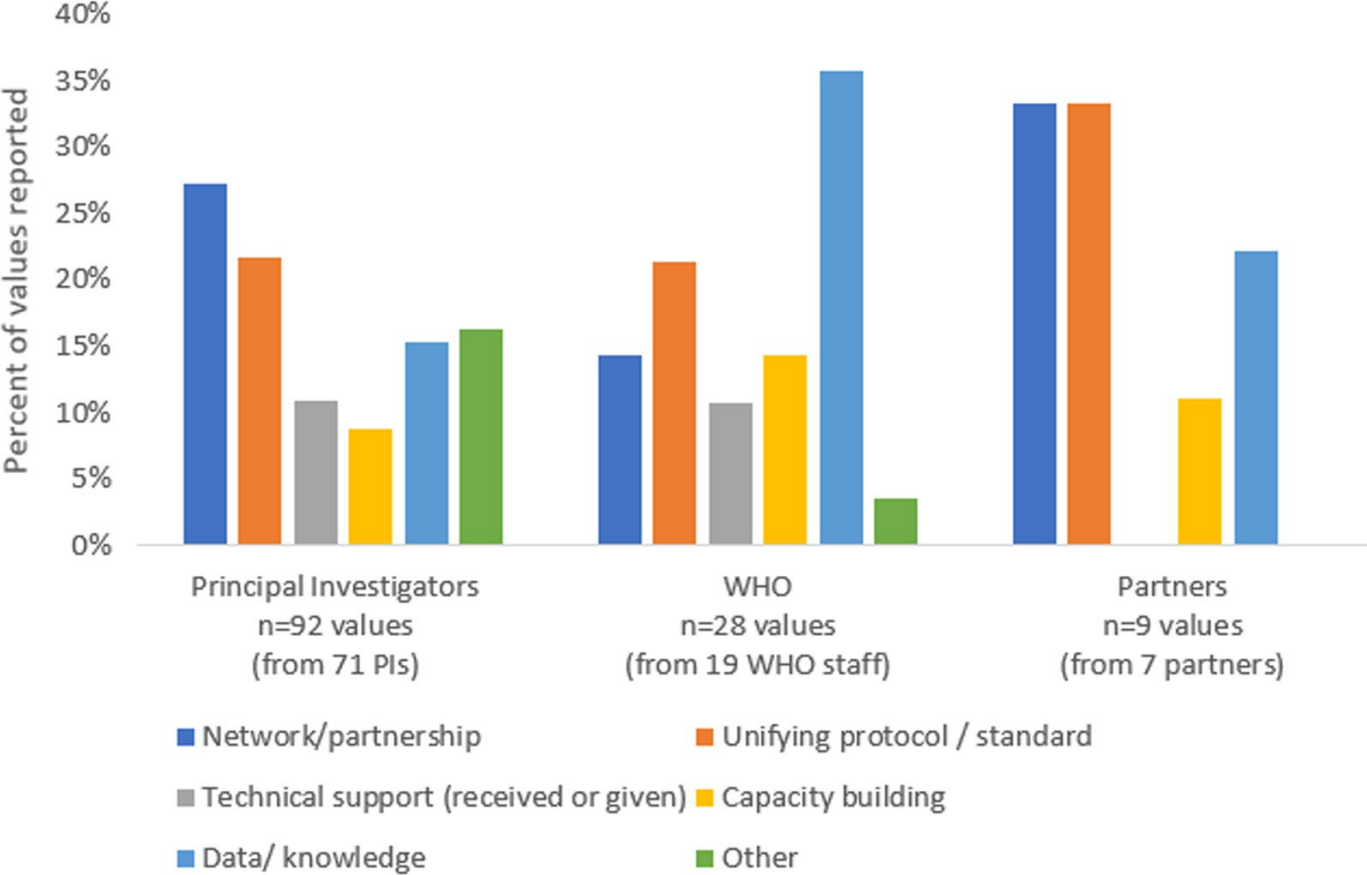
The biggest value of Unity Studies as reported by PIs (from survey and interviews), WHO Unity Studies focal points and stakeholders and external partners

Networking was the most frequently reported value by PIs (27% of the 92 responses from 71 PIs), followed by having a unifying protocol (22%) (Fig. 2). Regarding networking, PIs reported that engagement with the initiative brought about a highly valued network and community of practice including a range of subject matter experts representing national, regional and global experiences.

WHO and partner interviewees also highlighted the value of networking and protocols, as well as placing a high value on generating data and knowledge.

### The future

Two thirds of PI survey respondents (67%) strongly agreed that the Unity Studies initiative should become standard practice in the future, while 13 (19%) somewhat agreed, 10 (14%) were neutral and none disagreed.

There was also a high level of agreement among interviewees that the initiative should continue with further adjustments to streamline and facilitate more rapid implementation. A few exemplary comments on the future are shown below and provided insights for recommendations provided in Box 1A–C.

Insights on the Unity Studies approach in the future (relates to Box 1A):

*Only some countries should move forward here, those with a bit more capacity, it would be good to establish a network of such countries*. (WHO HQ)

*Find a way to engage HICs who have acted independently in the process; to engage them earlier rather than later*. (WHO RO)

*In future, there should be two sets of seroprevalence studies, at the beginning using residual samples (e.g. blood donors) and in later stages using population sampling*. (Partner)

*Need to focus on the inter=pandemic period—this is limited in most countries*. (Partner)

*There are a lot of other endemic diseases for which a similar initiative could become useful. WHO should look for scientists who have delivered and support them to conduct further studies across the continent*. (PI SEROPREV)

Management and technical support areas (relates to Box 1B):

*We need to get around sensitivities and confidentiality issues and increase visibility of these data*. (WHO HQ)

*Processes to ensure findings are used at global and regional level for policy-making should be established at an early stage or existing ones reinforced*. (WHO RO)

*Administrative processes in general need further standardization. One thing that should also be improved further is the clarity about the procurement of test kits, especially in terms of what is needed and how long it takes*. (WHO RO)

*In the future, there is the need to define processes to facilitate clearances and rapid uptake of studies—to get them moving.* (WHO RO)

*We should validate the results and diffuse them in the scientific community more widely as well as in the general public.* (Partner)

Capacity-building and networking (relates to Box 1C):

*The key is to be prepared to implement quickly, to have good capacity for quantitative methods, SOPs and ethics in place*. (WHO HQ)

*It would be good to have a complete training package*. (Partner)

*More capacity-building and communications in lay terms to explain to colleagues and the community about the importance of seroepidemiology and how results should be interpreted.* (WHO RO)

*We don’t have staff dedicated to research. This is one area that WHO will need to strengthen*. (WHO CO)

*The remedy here is all about education. There needs to be a message that operational research should be normalized*. (Partner)

## Discussion

The overwhelming majority of respondents surveyed and interviewed as part of this evaluation value the Unity Studies initiative and suggest it should be continued in the future. Even with this stance, PIs, WHO staff and partners noted several areas for improving readiness for rapidly implementing seroepidemiologic investigations in the face of a future pandemic.

### Using study findings for guiding policies and communications

Generating evidence that can validate or calibrate policies on preventing SARS-CoV-2 transmission or COVID-19 illness is one of the most valuable outcomes a Unity Study can have. About half of the Unity Studies PIs reported that findings were useful for informing or reinforcing PHSM and vaccination policies. This is an impressive finding, given that most studies were conducted in LMICs where PIs were faced with substantial technical and operational challenges. At the same time, this leaves space for improving in the future, especially for using findings to guide regional and global polices, which was limited. A higher proportion of PIs from LMICs reported that study findings were useful for informing PHSM policies and communications than those from HICs, suggesting that conducting seroepidemiologic studies is especially worthwhile in countries that may have weaker disease surveillance systems.

Using findings to guide communications efforts and contribute to generating knowledge of COVID-19 epidemiology was one of the most frequently reported uses of study findings. At the country level, study findings were shared with stakeholders, disseminated to the public and used to combat rumours and misinformation. However, a few PIs or governments felt it necessary to withhold findings of high seroprevalence to avoid negatively impacting PHSM or vaccination efforts.

At the global level, there is an array of good practices for disseminating study findings. Partnerships to compile and review findings were formed, existing platforms to catalogue studies leveraged, venues to discuss findings were established, and writing courses and collaborations to publish findings were made, all towards increasing the knowledge and skills base around SARS-CoV-2 seroepidemiology and towards setting a standard for the future [6–8].

However, dissemination alone does not necessarily translate to understanding and using findings. Multiple interviewees suggested that findings should be better synthesized and packaged so that they may be more easily understood and used by country decision-makers, stakeholder programmes and advisory bodies (Box 1C). Regional respondents reported limited human resources and skills to synthesize findings and share with their relevant advisory bodies. In the Unity Studies context, the role of interpreting and communicating findings lies at all levels, with the primary objective to inform local epidemiology and polices and secondarily to conduct comparisons and meta-analyses to inform country, regional and global epidemiology and polices—thus a central reason for developing generic protocols. To strengthen the use of findings for regional and global decision-making, increased capacity and expertise is needed, especially in the areas of data analytics and interpreting and communicating results for informing public health decisions and actions. Similarly, this would be an important part of a training curriculum to build further capacities at the national level (Box 1B).

Failure to consistently or completely report study details is also a barrier to using study findings. A recent meta-analysis of FFX studies showed a high and unexplained variance in infection rates that could be better understood if more details on the study context, methods and findings were provided [8]. The need for reporting standards has been articulated in several SARS-CoV-2 seroprevalence meta-analysis reports beyond Unity Studies [9–11] and is the basis for the recommendation to further detail reporting guidance for each protocol, such as the SOPs that have been developed for SEROPREV studies [12–14] (Box 1C).

Several interviewees were concerned that findings were often too complex to be used. As the virus evolved, vaccines were introduced and serologic assays were increasingly measuring different markers, it became more difficult to interpret and compare findings [15]. The initiative is well positioned to provide and update information briefs to support the better interpretation and use of findings over time. These briefs may also include guidance on considering biases and triangulating findings with other data sources to strengthen the evidence base (e.g. case or mortality data, vaccination coverage, other studies) (Box 1C).

Related to study design and implementation, respondents reported multiple factors that could limit study quality or comparability including defining participant eligibility, sampling representativeness, validation and cut-point setting of serologic kits, and deviating from protocols. The responsibility of study quality lies locally, with the study team, although the initiative played an important role in supporting countries and providing implementation guidance. Adding a systematic check-in or validation of studies’ adherence to protocols was suggested as an additional Unity Studies support role. If resources are constrained, this could be done for a sample of studies to have an indication of problem areas or for targeted studies to ensure priority studies are well implemented (Box 1A, 1B). Technical lessons from this pandemic will help establish a learning agenda for future capacity-building (Box 1C).

The overarching barrier to using study findings at all levels is that findings were not available in time to guide decisions. Though gaps in technical capacity contributed to delays, most delays were attributed to processes and management of study implementation and reporting.

### Unity Studies support areas and management

#### Technical support

Of the three support areas, the initiative’s provision of technical support received the highest level of satisfaction, with specific appreciation for support in adapting and reviewing protocols, advising on the suitability of serologic assays, data analysis and reporting results, and creating platforms for sharing findings and experiences. This is a notable finding given the emergency setting and limited number of staff engaged in the initiative.

The protocols were highly valued for providing standards and comparability. To facilitate faster turn-around times towards a final protocol, the initiative can enhance a training curriculum (including case studies) or produce learning resources that cover problematic areas that were encountered when supporting investigators in protocol adaption (e.g. sampling strategies and analysis plans) (Box 1C). The initiative may also consider defining a set of core variables and providing more periodic updates on the protocol templates to reflect the evolving characteristics of the pandemic.

On the data side, there was a call for increased support for data analysis and interpretation and for revisiting the use of the Go.Data data management tool. The Go.Data tool may be used by some in-country teams responsible for outbreak investigations, but PIs conducting Unity Studies were unfamiliar with the software and found it a frustrating time to have to become proficient in a new tool. These would also be important topics for a renewed training curriculum (Box 1C).

Of note is the initiative’s role and support for ethical clearance. At the country level, the process was generally found to be reasonable, as ERCs were experienced in reviewing operational research protocols and could expedite processes. PIs who experienced delays were in settings that lacked such experience or processes. National capacities can be strengthened by building on lessons from Ebola and other outbreak responses including the ability to distinguish between public health research and public health surveillance and to identify the appropriate ethical review processes, such as using preapprovals, waivers or expedited reviews [16] (Box 1C). Regarding the WHO ethical clearance required for Unity Studies support, like the local ERCs, regional ERCs are experienced in reviewing operational research protocols for their Member States, facilitating a smoother process compared to that of HQ. Overall, the role of WHO ethical clearance was brought up by several interviewees, not only because of the delays that were incurred but also questioning the added value it had, on top of country-level clearance. Besides urgently revisiting the specific role of WHO here, there is room to further shape WHO’s facilitation of ethical clearance of Unity Studies, including the possible preapproval of the generic protocols, reported to be helpful for FFX protocols (Box 1C).

#### Laboratory support

WHO guidance on selecting serologic test kits, identifying a preferred test kit for Unity Studies and facilitating their validation was greatly appreciated by study implementers and facilitated the comparability of results across studies [17]. However, delays in receiving the kits led to one of the evaluation’s lowest satisfaction scores. This is perhaps not surprising given the impact of the pandemic on global supply chains and considering that serologic assays were not considered a priority medical supply. Going forward, WHO should revisit their role in procuring and distributing test kits in terms of the value and cost to the organization and whether services could be improved by outsourcing (Box 1B).

In terms of laboratory capacity and support, increased resources are needed at the global and regional levels to manage the evaluation and decisions related to identifying preferred test kits, forums for dialogue and country support. External validation of test kit performance and laboratory methods may not be needed for all countries but could be considered for targeted countries or a sample of countries within each region. The process of identifying preferred test kits could be sped up by standardizing methodologies for evaluating test kit performance (Box 1C).

#### Financial support

Financial support received largely positive feedback and was noted to have substantially improved compared to the 2009 H1N1 pandemic. Successful fundraising efforts made it possible to fund studies, at least partially, in LMICs. However, some countries struggled to fill gaps of partial funding or had a difficult time spending funds before they expired. ROs reported cumbersome and inconsistent financial mechanisms that led to delayed fund disbursement and hiring of staff. Providing full financial support for key early studies, further refining emergency-mode funding streams and advocating for flexible spending windows are lessons to be taken from this pandemic (Box 1A and 1B).

#### Management and facilitation

The initiative brings a strong example of engaging and coordinating across all three levels of WHO. The shortfall is human resources, in terms of person-time and expertise in operational research, data analytics, laboratory science, and communication strategies—especially at the regional level. The toll of insufficient human resources came at a price of study delays and overstretching of the workforce. Mapping and securing the human resources needs for during and between pandemics will be a key step towards improved readiness and speed of a pandemic response (Box 1B).

Several WHO processes and procedures are not operating in emergency mode and put the organization’s reputation at risk by not being able to respond or be prepared in a timely manner. Areas of note include ethical clearance, staff hiring, fund disbursement and approval to disseminate time-sensitive technical guidance (Box 1B).

Even though a data-sharing agreement was a condition for receiving WHO Unity Studies support, this agreement was not binding, and receiving study data or findings was frequently delayed due to in-country approval processes or waiting for publication. Sharing findings from operational research in an emergency setting needs its own paradigm to allow for timely preliminary findings to be accounted for, while more time-consuming peer-review processes would take place in parallel. Recommendations for strengthening processes for receiving and dissemination data or findings are provided in Box 1B.

### Capacity-building

WHO positioned capacity-building not simply as a consequence of being part of the initiative but as a central and intentional aim to build national capacity to conduct operational research, enhance disease surveillance and contribute to research equity.

Capacity built through the initiative was highly appreciated, especially in the areas of research methodology and building partnerships. Opportunities to strengthen research skills came in the form of on-the-job training and peer-to-peer learning as part of technical support provided by the initiative. The multiple platforms established for sharing study findings and expertise served to build partnerships. And lastly, offering scientific writing courses not only was greatly appreciated and built capacity, but also facilitated the dissemination of findings.

Interviews revealed several weak spots in study design, management, implementation and reporting. Recommendations for addressing these, with the idea of pandemic preparedness and continuing to build national capacity to conduct operational research, are provided in Box 1C.

Identifying WHO staff at all levels and establishing technical partnerships to provide country support is a good model for the future, acknowledging that human resources and technical capacity-building at WHO ROs and COs would need to be enhanced.

### The future

Even with improvements in study implementation, reporting of findings and management, the needed study results may not come together in a timely or complete way. To optimize pandemic intelligence at the regional and global levels, one approach could be to enrol targeted countries and institutions with the capacity to conduct high-quality and timely operational research (including longitudinal studies) into a “fast-track” network (Box 1A). Engagement and capacity-building among LMICs should still be a focus, as well as securing engagement of HICs. This network could be sustained and closely managed in the inter-epidemic period and have established agreements to be activated immediately in the event of a new pandemic.

Related to a fast-track network, serosurveys using convenience sampling should be lined up to hit the ground running, to provide findings as early as possible in a pandemic. Earlier research has shown that estimates using convenience compared to random sampling are similar, with the cost being up to seven times higher when using population-based random sampling [7, 9, 18] (Box 1A). It will also be important to learn and share lessons from countries that were able to rapidly produce seroepidemiologic data, for example, from the United Kingdom’s adaptable research infrastructure that allowed it to rapidly redirect clinical research activities to such studies and use these for decision-making [19].

Participants shared ideas of what the Unity Studies initiative could look like in the future and suggested it should be sustained between outbreaks and pandemics. This could be done by mainstreaming and integrating it with existing surveillance programmes (Box 1A). The most obvious choice would be to integrate with influenza surveillance given the similarities in the epidemiology, monitoring and response (e.g. both are vaccine-preventable and have similar priority target groups) [20, 21]. It would also be important to explore integration, coordination and cross-learnings from other programmes that use serosurveys for monitoring and surveillance, such as HIV and parasitic and vaccine-preventable disease programmes [22–24], including the possibility to combine serosurvey efforts using multiplex assays [25, 26].

Finally, several investigators and stakeholders suggested broadening the scope of the Unity Studies initiative to include other diseases. This is certainly worth exploring further, especially for diseases caused by respiratory viruses and depending on where decisions fall on mainstreaming and integrating the initiative. In the meantime, strengthening the existing framework and mapping the future should be the top priority (Box 1A).

### Limitations

The evaluation had a few limitations. Study investigators were not always aware of the full extent to which their findings may have been used by decision-makers (e.g. several reported that findings were shared but that they were not engaged in the resulting decision-making process), leading to possible underestimation of usefulness. Regarding the online survey, bias related to incomplete PI contact information and nonresponse could have been introduced. Respondents were more likely to have received WHO support than nonresponders, but direction of bias is not clear, since extremely good and bad experience could be equally motivating reasons for completing the survey. Survey respondents and PI interviewees were more likely to come from LMICs; therefore, findings may overrepresent situations with limited capacities to manage and implement studies. Bias could have been introduced considering that some of the PI interviews were purposely selected by WHO RO focal points; however, the lack of a difference in the distribution of feedback between survey and interview investigators (Table 2) suggests that selection bias may be limited. Regarding stakeholder and partner interviews, both positive and negative feedback was provided, and there was no indication that any major stakeholders or partners were left out.

## Conclusions

The Unity Studies initiative was most valued by study investigators for, firstly, building a community of practice that provided access to experts and a platform for sharing findings, and secondly, for access to protocols that provided standards and a jump start on designing their studies. Study implementation and support received contributed to research equity, especially in LMICs. To better inform future pandemic responses, WHO should establish emergency-mode procedures to facilitate timely study implementation and continue to build national capacity to enable rapid study implementation and communication of findings in a format friendly to decision-makers.

## Box 1. Key recommendations from UNITY Studies evaluation







## Data Availability

Detailed interview and survey data are not publicly shared to protect confidentiality of respondents. Data are kept in the secure MMGH server environment for 5 years.

## Abbreviations

CO: Country office
COVID-19: Coronavirus disease 2019
ERC: Research Ethics Review Committee
EURO: Regional Office for Europe
FFX: First few cases
GOARN: Global Outbreak Alert and Response Network
HHT: Household transmission
HIC: High-income country
HQ: Headquarters
IMST: Incident Management Support Team
LMICs: Low- and middle-income countries
MMGH: MMGH Consulting
PHSM: Public health and social measures
PI: Principal investigator
RO: Regional office
SAGE: Strategic Advisory Group of Experts on Immunization
SARS-CoV-2: Severe acute respiratory syndrome coronavirus 2
SEROPREV: Population-based seroprevalence studies
